# Retraction: *Pseudomonas aeruginosa* mucinous phenotypes and *algUmucABD* operon mutant characteristics obtained from inpatients with bronchiectasis and their correlation with acute aggravation

**DOI:** 10.3389/fcimb.2025.1699040

**Published:** 2025-09-11

**Authors:** Frontiers Editorial Office

The journal retracts the July 29 2024 article cited above.

The authors retract the article cited above.

Following publication, the authors contacted the Editorial Office to request the retraction of the cited article, stating that errors in data entry and analysis were found and, after reanalysis, they were unable to reach statistically significant results. The findings reported in the article are no longer supported by the analysis. An investigation was conducted in accordance with Frontiers’ policies that confirmed this; therefore, the article has been retracted.

This retraction was approved by the Chief Editors of Frontiers in Cellular and Infection Microbiology and the Chief Executive Editor of Frontiers. The authors agree to this retraction.

